# Temporal Changes in Electromyographic Activity and Gait Ability during Extended Walking in Individuals Post-Stroke: A Pilot Study

**DOI:** 10.3390/healthcare9040444

**Published:** 2021-04-10

**Authors:** Kazuki Fujita, Yasutaka Kobayashi, Masahito Hitosugi

**Affiliations:** 1 Department of Rehabilitation, Faculty of Health Science, Fukui Health Science University, Fukui 910-3190, Japan; yasutaka_k@fukui-hsu.ac.jp; 2 Department of Legal Medicine, Shiga University of Medical Science, Shiga 520-2192, Japan; hitosugi@belle.shiga-med.ac.jp

**Keywords:** electromyography, stroke, temporal endurance, muscle activity

## Abstract

Abnormal gait, particularly in patients with stroke, causes neuromuscular fatigue. We aimed to clarify temporal changes in gait performance and lower limb muscle activity during extended walking in people with stroke hemiplegia. Twelve adults with stroke and eleven healthy controls performed an extended trial involving 20-min continuous walk at a comfortable speed. The primary outcome was electromyography amplitude during the trial and secondary outcomes were walking performance and the instantaneous mean frequency of electromyography during the trial. Data at 1, 6, 12, and 18 min after initiating walking were compared. Performance during extended walking in people with stroke was maintained over time. The electromyography amplitude decreased in the tibialis anterior during the pre-swing phase and increased in the rectus femoris during the single-support phase over time; these changes were similar on the paretic and nonparetic sides. Instantaneous mean frequency decreased over time on the nonparetic side in the tibialis anterior and on the paretic side in the rectus femoris. Healthy subjects did not show any changes over time. The changes in muscle activity in patients with stroke differed between the paretic and nonparetic sides, muscle type, and gait phase; walking performance was maintained despite being affected by neuromuscular fatigue.

## 1. Introduction

Stroke mortality has declined rapidly worldwide [1]; however, stroke is the third leading cause of disability [2]. Although age-adjusted stroke mortality has declined in Japan as well, stroke remains one of the leading causes of death and disability [3]. Of the various disorders that occur after a stroke, fatigue is a common sequela and affects up to 72% of patients [4,5,6]. In addition, fatigue and gait are symptoms that patients with stroke themselves preferentially choose to improve [7]. Therefore, there are many patients with stroke for whom fatigue is a problem, and it is important to improve poststroke fatigue and walking problems for better participation in society and improved quality of life. To date, no studies have investigated lower limb fatigue associated with extended walking in patients with stroke.

Rudroff T et al. [8] proposed the definition of fatigue as the decrease in physical and/or mental performance that results from changes in central, psychological, and/or peripheral factors. One of the causes of fatigue perception is a neuromuscular origin that occurs due to physiological changes in the central and peripheral motor pathways, resulting in physical changes (e.g., muscle atrophy) and impaired functional abilities [9,10]. For example, in a study, failure in the voluntary activation of the tibialis anterior muscle following a sustained submaximal fatigue task in stroke patients was more evident on the paretic side than on the nonparetic side [11]. This failure of force production occurs at any one or combinations of the various sites along the motor pathway from the central nervous system through to the intramuscular contractile apparatus, resulting in a significant impact on the physical activity of a person affected with stroke [9].

The origin of neuromuscular fatigue is divided into central and peripheral; central fatigue refers to a progressive contraction-induced reduction in the ability to activate a skeletal muscle voluntarily at spinal cord or supraspinal level, while peripheral fatigue refers to a loss of force due to failure in neuromuscular signal transmission or in the contractile apparatus of the muscle fibers [9,12,13]. In previous studies, electromyographic (EMG) evaluation has been used to distinguish between these two neuromuscular fatigue factors. A decrease in EMG amplitude reflects a decrease in the number of motor units participating in the activity or a decrease in firing frequency, i.e., the effects of central fatigue [14]. A reduction in EMG power frequency reflects, at least in part, a reduction in the conduction velocity of the electrical signal along the muscle fiber membrane, i.e., the effects of peripheral fatigue [9]. Many studies investigating neuromuscular fatigue in patients with stroke using such electrophysiological techniques have been a fatigue task for muscle contraction due to voluntary or electrical stimulation [15,16,17]. On the other hand, in patients with stroke, the increased muscle coactivation and decreased motor control appear during walking [18,19], suggesting that such abnormal gait causes neuromuscular fatigue [9].

Very few studies have investigated time-dependent fatigue during walking. In one such study, people with hemiplegia had a reduced walking distance for each subsequent 2-min period of the 6 min walk test (6MWT), and those with poor functional capacity had worsened stance time asymmetry [4]. In this study, the impact of neuromuscular fatigue on gait was unknown because of the lack of electrophysiological evaluation. In addition, although many intervention studies by gait training for patients with stroke are conducted continuously for 20 min or more [20], the mechanism of neuromuscular fatigue that occurs in gait tests longer than 6 MWT has not been clarified. Reports related to extended walking indicate that postural stability is reduced in people with hemiplegia after walking for 18 min compared to those walking for 6 min, indicating that people with hemiplegia may exhibit a decline in walking performance over time during longer functional walking tests [5]. Following a stroke, fatigue-induced suboptimal output from the motor cortex, either due to inadequate input from sites upstream, or due to a decrease in motor cortex excitability [3] that might otherwise result in extended walking performance is negatively affected. In addition, skeletal muscle atrophy and muscle fiber type II shifts are induced after stroke [21], and peripheral fatigue may also affect extended walking. Furthermore, the mechanism of muscle fatigue that differs between paretic and nonparetic sides [8], differences in fiber type ratios in different muscles [22], and walking muscle activity patterns that differ from those of an unaffected person [23] are also considered to impact the performance of extended walking. Therefore, it is important to investigate changes in muscle activity in various parts during extended walking by surface electromyography to adjust the walking training time in consideration of neuromuscular fatigue and determine the target parts of muscle endurance training.

The present study aimed to clarify temporal changes in gait performance and lower limb muscle activity during extended walking in people with stroke hemiplegia. The hypothesis was that muscle activity would decrease with central or peripheral fatigue only on the paretic side, and walking speed would decrease.

## 2. Materials and Methods

### 2.1. Subjects

In this pilot cross-sectional study, we recruited subjects from among the 22 stroke patients who were hospitalized between April 2018 and October 2019 solely for the purpose of receiving intensive physiotherapy for movement disorders or spasticity due to stroke sequelae. The inclusion criteria were as follows: (i) pyramidal tract damage due to unilateral cerebral lesions in poststroke patients, (ii) a period of at least 6 months since the onset of stroke, (iii) ability to walk independently for >20 min continuously and (iv) no pain reported in the body during walking. The exclusion criteria were as follows: (i) history of lower limb joint surgery, (ii) Mini-Mental State Examination score of <24 points, and (iii) treatment received for cardiovascular disease and/or respiratory disease. Control data were collected from eleven healthy subjects whose age, height, and weight were within the range of mean plus twice the standard deviation in patients with stroke (Table 1). All subjects provided written informed consent for participation. This study was approved by the Ethical Review Committee of Nittazuka Medical Welfare Center (approval no.: Nittazuka Ethics 29-114).

### 2.2. Assessments

Subjects performed an extended walking trial that involved them walking continuously for 20 min at a comfortable speed on the first day of hospitalization. This trial used a 20-m horizontal straight walkway, with the subjects walking lengths back and forth. A video camera (HD Pro WebCamera, Logicool, Inc., Tokyo, Japan) with a sampling frequency of 30 Hz was set 5 m laterally offset at the midpoint of the walkway. Subjects with a high risk of falling due to toe dragging during the swing phase were allowed to use an ankle foot orthosis; however, to minimize their impact on the results, we used ORTOP^®^, which has a short, thin, and light plastic body [24]. Therefore, this ankle foot orthosis has a weak fixing force and the ankle joint moves slightly during walking. The criteria for discontinuation of a trial were: if subjects exceeded 70% of their maximum heart rate (220-age; HR), or the rating of perceived exertion (RPE) was >6 points [25,26].

The primary outcome was the EMG amplitude during the trial, and the secondary outcomes were the walking performance and instantaneous mean frequency (IMNF) of the EMG during the trial. TELEmyo DTS (Noraxon Inc., Scottsdale, AZ, USA) was used for EMG recording during gait. Sampling frequency was 1500 Hz, and the bandpass filter was set at 10–500 Hz. EMG was performed for the following muscles bilaterally in patients with stroke: tibialis anterior, soleus, rectus femoris, and biceps femoris. Although healthy gait is often characterized as symmetric, there may be subtle asymmetric patterns in both kinematic and kinetic measurements [27]; therefore, in healthy subjects, the EMG data were collected from the dominant lower limb. Skin impedance was reduced to ≤20 kΩ using alcohol-soaked cotton swabs and an abrasive cream (skinPure, Nihon Kohden Co., Ltd., Tokyo, Japan). A Dual EMG Electrode (EM-272, Noraxon) with an interelectrode distance of 2 cm between Ag–AgCl electrodes was applied to the treated skin area [23]. To identify the gait phase, foot switches (Noraxon Inc.) were placed on both feet [18]. MyoSync and Sync Light (Noraxon Inc.) were used to synchronize all instruments and ensure alignment of the time frames.

### 2.3. Data Analysis

MR3 software (Noraxon Inc.) was used for analyzing the EMG waveforms. The EMG waveform was full-wave rectified and passed through a 20-Hz low-pass Butterworth filter [28]. The analyzed periods began at 1 min (baseline), 6 min, 12 min, and 18 min after the start of walking, and each period covered three continuous gait cycles. Initial contact was defined as the time of electric potential input from the foot switch on the paretic side of the body and was determined using synchronized video cameras, with monitoring for abnormal electric potentials from the foot switch due to leg-dragging during the swing phase. The duration of each gait cycle was temporally normalized after considering one gait cycle to be 100%. Next, the arithmetic mean for the three gait cycles was obtained, and 1000-point amplitudes were calculated at intervals of 0.1%. Finally, the amplitude of each muscle was normalized by the corresponding muscle peak value in the entire analyzed period.

The double support after paretic initial contact (i.e., loading response on the paretic side and pre-swing on the nonparetic side), single support on the paretic side (i.e., swing on the nonparetic side), double support before paretic swing (i.e., pre-swing on the paretic side and loading response on the nonparetic side), and swing on the paretic side (i.e., single support on the nonparetic side) for each gait phase were identified from the footswitch data on both sides [29], and the EMG mean amplitude and time period at each gait phase were calculated. Step length was measured using still images extracted from the video data when subjects passed through the intermediate points on the walkway [18]. Gait velocity was calculated using the measured stride length and cadence. The asymmetry index was calculated from the step length and the swing periods [18].

To extract information relating to changes in frequency and time from the EMG signal, frequency analysis was performed using continuous wavelet transform (CWT) [30]. CWT was performed on raw waveforms in each analysis period, using the Morlet wavelet as the mother function [22]. The output of CWT is *x*-axis for time (gait cycle), *y*-axis for frequency (set to 10–500 Hz), *z*-axis for power (magnitude), and the IMNF of each analysis period was calculated using the following equation [28,30]:

(1) $$IMNF=\;\frac{\sum_{j=1}^{M}f_{j}W\left(f_{j},t\right)}{\sum_{j=1}^{M}W\left(f_{j},t\right)}$$

### 2.4. Statistical Analysis

Corresponding to the normality and homoscedasticity of the data performed on the collected data, differences between groups after 1 min from the start of walking were analyzed using the independent *t*-test, Welch’s *t*-test, or Mann–Whitney U-test (people with stroke vs. healthy subjects) and the one-factor ANOVA with non-repeated measures or Kruskal–Wallis test (paretic side vs. nonparetic side vs. dominant side in healthy subjects). Differences between data from the four periods (1, 6, 12, and 18 min) within the groups were analyzed using one-factor ANOVA with repeated measures or the Friedman test corresponding to the normality test. A post-hoc analysis was performed using the Bonferroni post-hoc multiple comparison test or Scheffé test.

Cohen’s d was used to assess effect size (ES) [31]. For performing these statistical analyses, BellCurve for Excel (Social Survey Research Information Co., Ltd., Tokyo, Japan) was used, with a significance level set at 5%. The sample size was determined using G* Power 3.1 (Heinrich-Heine-University, Düsseldorf, Germany) (ES (f): 0.3; significance level: 5%; statistics power: 80%; correlation among repeated measures: 0.7; calculated total sample size: 11) [32].

## 3. Results

Fifteen of the 22 patients met the inclusion criteria; thereafter, three patients who met the exclusion criteria were excluded and finally, a total of 12 patients participated in the study (Table 1). All subjects completed 20 min of continuous walking without reaching the discontinuation criteria for HR and RPE during the extended walking test.

### 3.1. Gait Performance

Comparisons of the baseline (1 min after starting walking) between subjects with stroke and healthy subjects showed that gait velocity (*p* < 0.001, t = 7.70), stride length (*p* < 0.001, t = 7.61), cadence (*p* < 0.001, t = 4.85), and single-support phase period (*p* < 0.001, t = 5.58) were significantly lower in those with stroke, while pre-swing phase period (*p* = 0.040, t = 2.26), swing phase period (*p* = 0.006, t = 3.22), and the asymmetry index for swing phase period (*p* < 0.001, t = 6.43) were significantly larger in those with stroke (Table 2).

With stroke, gait velocity (*p* = 0.004, t = 3.80, ES = 0.36) and stride length (*p* = 0.004, t = 3.79, ES = 0.46) were significantly increased 18 min after the start of walking compared with baseline. In healthy subjects, the spatiotemporal parameters were not significantly different between each period during the extended walk (Table 2).

### 3.2. EMG Amplitude

In the tibialis anterior at baseline, the EMG amplitude during pre-swing phase on the paretic side was significantly greater than that on the nonparetic side (*p* = 0.010, t = 3.19) and the legs of healthy subjects (*p* < 0.001, t = 4.89), and the amplitude during single-support phase on the paretic (*p* = 0.048, t = 2.54) and nonparetic sides (*p* = 0.002, t = 3.81) was significantly greater than that in the legs of healthy subjects. In the soleus at baseline, the amplitude during loading response phase on the paretic side was significantly greater than on the nonparetic side (*p* = 0.009, t = 3.23) and in the legs of healthy subjects (*p* < 0.001, t = 5.36), and the amplitude during single-support phase on the paretic side was significantly less than that in the legs of healthy subjects (*p* = 0.044, t = 2.58), and the amplitude during pre-swing phase on the nonparetic side was significantly greater than that in the legs of healthy subjects (*p* = 0.019, t = 2.91). In the biceps femoris at baseline, the amplitude during single-support phase on the nonparetic side was significantly greater than that in the legs of healthy subjects (*p* = 0.034, t = 2.69) (Table 3).

**Figure 1:**
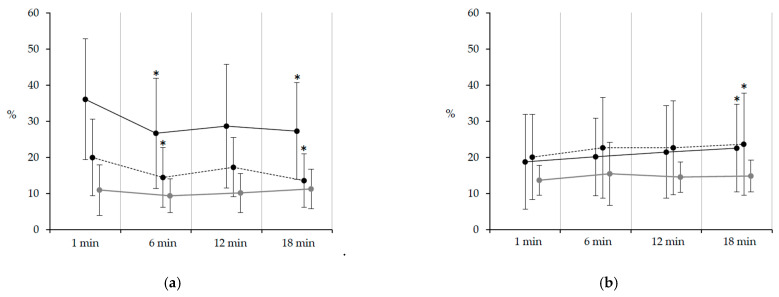
Temporal changes in electromyography amplitude during the extended walking test. Values are expressed as mean (standard deviation). (**a**) Tibialis anterior during the pre-swing phase; (**b**) rectus femoris during the single-support phase. The solid line represents the paretic side, the dotted line represents the nonparetic side, and the gray line represents healthy subjects. The EMG amplitude in the tibialis anterior during the pre-swing phase on the paretic side significantly decreased at 6 min and 18 min, and on the nonparetic side significantly decreased at 6 min and 18 min compared with that at 1 min. The amplitude in the rectus femoris during the single-support phase on the paretic side significantly increased at 18 min, and on the nonparetic side significantly increased at 18 min compared with that at 1 min.

The EMG amplitude in the tibialis anterior during the pre-swing phase on the paretic side significantly decreased at 6 min (*p* = 0.024, t = 3.08, ES = 0.59) and 18 min (*p* = 0.041, t = 2.88, ES = 0.58), and on the nonparetic side also significantly decreased at 6 min (*p* = 0.012, t = 3.35, ES = 0.58) and 18 min (*p* = 0.003, t = 3.87, ES = 0.69) compared with baseline. The amplitude in the rectus femoris during the single-support phase on the paretic side significantly increased at 18 min (*p* = 0.029, t = 3.02, ES = 0.30), and on the nonparetic side also significantly increased at 18 min (*p* = 0.034, t = 2.98, ES = 0.28) compared with baseline (Figure 1). In healthy subjects, the amplitudes in each muscle were not significantly different between periods during the extended walk.

### 3.3. EMG Frequency

**Figure 2:**
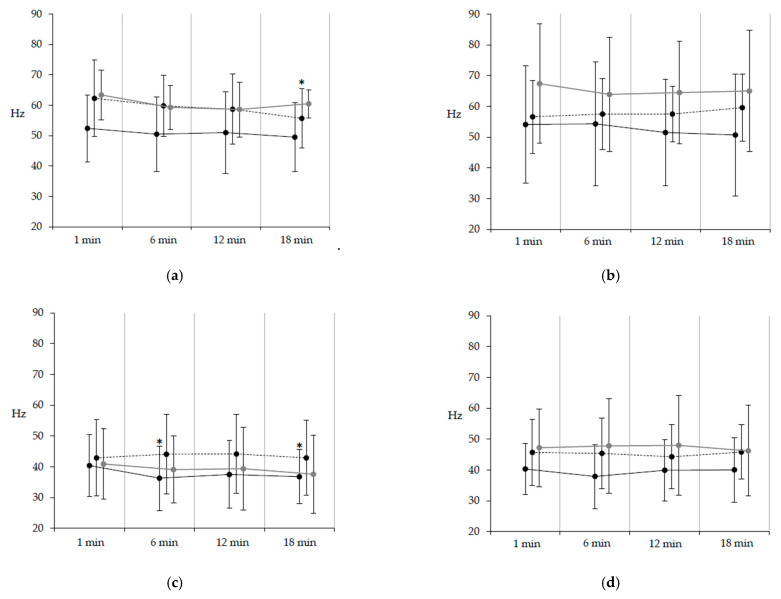
Temporal changes in the instantaneous mean frequency (IMNF) during the extended walking test. Values are expressed as mean (standard deviation). (**a**) Tibialis anterior; (**b**) Soleus; (**c**) Rectus femoris; (**d**) Biceps femoris. The solid line represents the paretic side, the dotted line represents the nonparetic side, and the gray line represents healthy subjects. The IMNF of the tibialis anterior on the nonparetic side significantly decreased at 18 min compared with that at 1 min. The IMNF of the rectus femoris on the paretic side significantly decreased at 6 min and 18 min compared with that at 1 min.

The IMNF of each muscle did not differ significantly between groups at baseline. The IMNF of the rectus femoris on the paretic side significantly decreased at 6 min (*p* = 0.004, t = 3.72, ES = 0.40) and 18 min (*p* = 0.019, t = 3.19, ES = 0.37) compared with baseline. The IMNF of the tibialis anterior on the nonparetic side significantly decreased at 18 min (*p* = 0.039, t = 2.91, ES = 0.59) compared with baseline (Figure 2).

## 4. Discussion

The physical characteristics of the subjects with stroke in this study consisted of mild to moderate movement disorders, muscle weakness, and spasticity. In the subjects with stroke, the gait velocity and stride length increased after 20 min from the start of walking; however, other gait parameters did not change over time. The EMG amplitude decreased in the tibialis anterior during the pre-swing phase and increased in the rectus femoris during the single-support phase, these changes being similar on the paretic and nonparetic sides. Meanwhile, a downward shift in EMG frequency was observed only in the nonparetic side tibialis anterior and only in the paretic side rectus femoris. All the data for the healthy subjects remained unchanged over time.

In a previous study [4], walking performance decreased during the 6MWT, but in this study, walking performance was maintained despite a longer walking time. For the subjects with hemiplegia, the change in walking time from 6 to 18 min represented a shift from light to hard physical exertion [5]. It is probable that the subjects in this study experienced fatigue because RPE increased over time. Nevertheless, HR was stable after 6 min and walking performance was maintained. The increase in RPE may be attributable to the presence of stroke-specific impairments (e.g., muscle weakness or spasticity) [33]. The contradiction between increased RPE and maintenance of walking performance may have been influenced by the changes in muscle activity identified in this study.

The pre-swing phase on the paretic side was greater than that in healthy subjects. The strength of the ankle dorsiflexors is important to ensure foot clearance during gait in people with hemiplegia [34]. However, in paretic muscles, the output of each motor unit is reduced, so more motor units need to be activated to reach the required level of force [35]. In fact, since the subjects with stroke in this study had weak ankle dorsiflexor strength (Table 1), to achieve a satisfactory pre-swing may have necessitated an increase in the number of motor units and frequency of firing in the tibialis anterior. This increased amplitude of the tibialis anterior on the paretic side decreased over time. On the other hand, a downward shift in frequency that would have indicated peripheral fatigue [30] was not observed. Previous studies investigating the effect of stroke on neuromuscular fatigability using voluntary contractions, as a means to induce fatigue, reported increased central fatigue (evidenced by impaired voluntary muscle activation) and reduced peripheral fatigue (evidenced by decreased EMG spectral shift) on the paretic side compared with the nonparetic side in controls [9,11,15]. This reaction, specific to the paretic side, which was also observed on the tibialis anterior in present study, is considered to be a protective control mechanism for avoiding transmission deficiency in peripheral muscle, optimizing muscle output, and minimizing energy loss [9,36].

Meanwhile, in the tibialis anterior on the nonparetic side, a downward shift in frequency showing peripheral fatigue was observed in addition to an amplitude decrease over time. In subjects with stroke in this study, the single-support phase on the nonparetic side (i.e., the swing phase of the paretic side) was longer than in healthy subjects at baseline, and the mean amplitude for the tibialis anterior on the nonparetic side during that phase was higher than in healthy subjects (controls). Therefore, the tibialis anterior on the nonparetic side was required to contract for a longer period and to a greater extent. This sustained muscle contraction is more likely to cause muscle fatigue than more intermittent contraction [9], and the tibialis anterior on the nonparetic side may consequently have been more susceptible to peripheral fatigue. Therefore, the tibialis anterior tends to have less endurance on both the paretic and the nonparetic sides, suggesting that it is a target for muscle endurance training. Moreover, since the fatigue mechanism is different on the paretic and nonparetic sides, the approach method will be different.

The amplitude in the rectus femoris on both sides in subjects with stroke increased over time during the single-support phase. The increase in electrical activity during the fatigue task is due to recruitment of motor units to compensate for the decrease in force of contraction occurring in the fatigued muscle fibers [37]. Since the proportion of type II fibers in the rectus femoris is much larger than that in other measured muscles (tibialis anterior, soleus, and biceps femoris) [22], it may have been susceptible to muscle fatigue. Furthermore, the rectus femoris on the paretic side also exhibited a downward shift in frequency. It has been shown that this reaction, a downward shift in frequency despite an increase in amplitude, occurs when motor units are synchronized [38]. Increased levels of synchronization may promote higher initial discharge rates by motor neurons during rapid contractions, appearing to enhance the rate of increase in force [39]. Therefore, this effect may have contributed to the increase or maintenance of walking performance by synchronizing the motor units in the knee extensor muscles on the paretic side. Patients with stroke have increased perceptual fatigue at early stage during extended walking; moreover, longer duration of walking training can promote neuroplasticity.

The limitation of this study is that it remains unclear how the changes in muscle activity over time affected the actual joint kinematics. For example, the decrease in tibialis anterior activity over time may have resulted in decreased foot clearance by decreasing the ankle dorsiflexion angle, even though walking performance was maintained over time in subjects with stroke. It has been reported that a decrease in ankle dorsiflexion caused by continuous gait tests in people with hemiplegia is compensated for by a concomitant increase in hip and knee flexion angles during swing [40]. Older adults switch to walking faster with longer strides to overcome the feeling of fatigue-induced physical discomfort as quickly as possible [41]. In this study, there were no changes in muscle activity associated with the phenomena observed in these previous studies, but the activity of the hip flexors (such as the iliopsoas), which could not be measured by surface EMG, may have affected the maintenance of walking performance. Alternatively, the effect of changes in muscle activity due to extended walking on the changes in walking performance may be small. In this study, 4 out of the 12 subjects with stroke used an ankle foot orthosis during extended walking. However, as the ankle foot orthosis was small and lightweight, the fixing force was weak. Although the ankle foot orthosis assisted the dorsiflexion of the ankle joint, neuromuscular fatigue was observed in the tibialis anterior muscle on the paralyzed side. In other words, it is probable that similar results would have been obtained for the tibialis anterior muscle even if the four patients did not use the brace. In addition, since the fixing force of the ankle joint is weak, the influence on the muscles of the knee joint is considered to be small. Therefore, although the impact on the results cannot be completely denied, the impact is likely to have been small. In addition, 10 of 12 subjects in this study had putamen hemorrhage. All the subjects presented with hemiplegia due to pyramidal tract damage, but because the striatum is involved in motor control [42], the results of this study may be particularly relevant to patients with putamen hemorrhage.

## 5. Conclusions

We examined changes over time in walking performance and lower limb muscle activity during an extended continuous walk of 20 min in people with stroke hemiplegia. Muscle activity changes differed between the paretic and nonparetic sides, muscle type, and gait phase, and were affected by several fatigue mechanisms. In subjects with stroke, gait velocity and stride length increased, and other gait parameters did not worsen, despite an apparent increase in fatigue over time. The results of this study will contribute to the setting of walking training time in consideration of the occurrence of neuromuscular fatigue and changes in neuromuscular activity patterns with over time, and the determination of the target site for muscle endurance training in patients with stroke.

## Figures and Tables

**Table 1:** Characteristics of subjects.

		With Stroke (*n* = 12)	Healthy (*n* = 11)	*p*
Age	(years)	54.7 ± 9.5	55.5 ± 7.9	NS
Height	(cm)	168.9 ± 7.9	168.5 ± 8.5	NS
Weight	(kg)	66.8 ± 12.6	65.0 ± 10.6	NS
Sex	(female/male)	3/9	3/8	NS
Type of stroke	Putaminal hemorrhage Subcortical infarction Basal ganglia infarction	*n* = 10 *n* = 1 *n* = 1	N/A	
Months since stroke onset		60.4 ± 25.4	N/A	
Paretic side	(L/R)	5/7	N/A	
Assistive device	(None/AFO)	8/4	N/A	
Fugl–Meyer assessment LE		21.3 ± 3.8	N/A	
Motricity index				
	Hip flexors	25 (8)	33 (0)	<0.05
	Knee extensors	25 (8)	33 (0)	<0.05
	Ankle dorsiflexors	19 (5)	33 (0)	<0.05
Modified Ashworth scale				
	Hip flexors	1 (1)	N/A	
	Knee extensors	1.5 (1)	N/A	
	Ankle plantar flexors	2 (2)	N/A	

Values are presented as mean ± standard deviation or median (quartile deviation). *p* values refer to comparison between subjects with stroke and control group (*t*-test, Chi-squared test, or Mann–Whitney U-test). A score of 1+ in the Modified Ashworth scale was assigned as 2 and scores of ≥2 were revised upward by 1. CI, cerebral infarction; ICH, intracerebral hemorrhage; LE, lower extremity; AFO, ankle foot orthosis.

**Table 2:** Gait performance changes over time during the extended walk test.

		With Stroke (*n* = 12)				Healthy (*n* = 11)			
		1 min	6 min	12 min	18 min	1 min	6 min	12 min	18 min
Gait velocity	(m/sec)	0.65 ^a^	0.68	0.69	0.73 ^†^	1.25 ^a^	1.25	1.28	1.29
		(0.22)	(0.23)	(0.24)	(0.25)	(0.15)	(0.17)	(0.16)	(0.15)
Stride length	(cm)	83.7 ^a^	87.8	89.1	92.0 ^†^	132.2 ^a^	134.5	135.5	135.6
		(16.3)	(18.0)	(19.3)	(19.7)	(13.9)	(15.3)	(15.6)	(15.7)
Cadence	(steps/min)	90.8 ^b^	90.5	91.4	93.2	113.2 ^b^	111.6	113.3	113.9
		(15.4)	(15.5)	(15.8)	(16.5)	(3.8)	(3.6)	(3.1)	(3.9)
LR period	(% GC)	14.4	14.6	14.6	14.2	13.7	14.5	13.7	13.4
		(3.5)	(4.0)	(4.3)	(5.1)	(1.6)	(1.8)	(2.0)	(1.8)
SS period	(% GC)	28.0 ^b^	28.3	28.0	28.5	36.6 ^b^	36.0	36.2	36.8
		(4.9)	(4.6)	(5.0)	(4.4)	(2.1)	(1.8)	(2.2)	(2.3)
PSw period	(% GC)	17.1 ^b^	17.5	17.6	17.4	13.5 ^b^	13.8	13.7	13.1
		(5.1)	(4.8)	(4.7)	(4.2)	(2.1)	(1.9)	(2.0)	(2.5)
Sw period	(% GC)	40.4 ^b^	39.6	39.8	39.8	36.2 ^b^	35.8	36.3	36.6
		(4.3)	(4.3)	(4.2)	(4.0)	(1.5)	(1.7)	(1.8)	(1.9)
AI for Sw period	(%)	59.2 ^b^	58.4	58.9	58.4	49.7 ^b^	49.8	50.1	49.9
		(5.0)	(4.8)	(4.9)	(4.1)	(0.7)	(0.6)	(0.8)	(0.7)
AI for step length	(%)	51.2	52.8	52.7	52.6	50.3	50.4	50.0	50.0
		(5.3)	(4.2)	(3.8)	(4.0)	(0.3)	(0.4)	(0.5)	(0.6)
Heart Rate	(beats/min)	83.1	103.4 ^†^	110.1 ^†^	107.8 ^†^	84.0	93.9 ^†^	97.6 ^† ‡^	100.2 ^† ‡^
		(7.8)	(11.6)	(10.1)	(16.3)	(6.7)	(5.1)	(6.2)	(8.7)
RPE		0	1	2 ^†^	3 ^†^	0	0.5	1 ^†^	2 ^† ‡^
		(0)	(0.5)	(1.5)	(2.5)	(0)	(0.75)	(1)	(1)

The gait parameters and the heart rate are presented as mean (standard deviation). The RPE is presented as median (quartile deviation). Comparison of values at 1 min between the stroke and control groups: ^a^ *p* < 0.05 (*t*-test); ^b^ *p* < 0.05 (Welch’s *t*-test). Comparison over time within the groups: ^†^ for 1 min, *p* < 0.05; ^‡^ for 6 min, *p* < 0.05 (Bonferroni test or Scheffé test). LR, loading response; SS, single support; PSw, pre-swing; Sw, swing; AI, asymmetry index; RPE, rating of perceived exertion.

**Table 3:** Electromyography amplitude changes over time during the extended walking test.

		Paretic Side (*n* = 12)				Nonparetic Side (*n* = 12)				Healthy (*n* = 11)			
		1 min	6 min	12 min	18 min	1 min	6 min	12 min	18 min	1 min	6 min	12 min	18 min
TA	LR	26.2	25.6	22.9	28.6	37.9	38.3	41.1	43.3	38.4	37.5	33.6	35.3
		(12.8)	(10.8)	(10.7)	(15.1)	(16.1)	(17.9)	(14.3)	(15.8)	(10.0)	(9.3)	(6.8)	(9.5)
	SS	16.3 ^b^	11.3	11.5	12.8	21.7 ^c^	21.6	21.7	17.5	5.4 ^b c^	5.3	5.1	6.5
		(14.0)	(9.4)	(8.0)	(8.1)	(10.2)	(14.7)	(12.4)	(8.9)	(2.3)	(2.3)	(1.8)	(2.9)
	PSw	36.1 ^a b^	26.7 ^†^	28.7	27.3 ^†^	20.0 ^a^	14.5 ^†^	17.3	13.6 ^†^	11.0 ^b^	9.4	10.2	11.3
		(16.7)	(15.2)	(17.1)	(13.4)	(10.6)	(8.3)	(8.2)	(7.4)	(7.0)	(4.7)	(5.4)	(5.5)
	Sw	27.7	23.2	25.3	26.6	26.5	22.9	23.9	24.8	22.4	21.0	22.2	24.0
		(13.0)	(9.5)	(10.6)	(11.3)	(11.6)	(11.2)	(9.1)	(10.1)	(8.2)	(7.8)	(8.8)	(9.7)
Sol	LR	30.9 ^a b^	28.5	30.5	32.1	17.9 ^a^	17.4	15.5	16.3	8.8 ^b^	10.1	9.1	10.4
		(12.1)	(8.5)	(8.3)	(10.2)	(10.5)	(12.0)	(10.3)	(10.6)	(5.6)	(9.2)	(5.5)	(7.7)
	SS	24.6 ^b^	21.7	23.1	25.1	29.1	25.9	26.1	29.8	37.5 ^b^	35.7	37.8	38.3
		(13.1)	(9.3)	(9.6)	(11.1)	(10.3)	(8.6)	(10.2)	(10.8)	(12.3)	(11.5)	(11.2)	(11.6)
	PSw	14.2	14.0	15.7	15.8	23.1 ^c^	24.5	20.8	24.5	9.5 ^c^	8.9	9.5	9.3
		(9.1)	(8.8)	(9.7)	10.8)	(16.0)	(16.0)	(15.6)	(15.3)	(5.7)	(5.2)	(7.2)	(5.0)
	Sw	12.6	10.3	11.0	13.1	10.2	11.2	7.9	8.9	8.2	8.7	10.6	9.5
		(8.1)	(6.0)	(7.0)	(9.5)	(8.1)	(9.6)	(7.5)	(7.0)	(4.5)	(4.3)	(7.1)	(5.4)
RF	LR	32.8	34.5	32.5	34.9	34.3	31.8	36.8	35.5	39.1	40.1	42.4	47.0
		(15.9)	(12.6)	(14.9)	(17.0)	(12.2)	(13.1)	(14.9)	(14.9)	(16.7)	(16.5)	(16.9)	(20.4)
	SS	18.8	20.2	21.5	22.6 ^†^	20.1	22.7	22.7	23.7 ^†^	13.7	15.5	14.6	14.9
		(13.1)	(10.7)	(12.8)	(12.1)	(11.8)	(13.9)	(13.0)	(14.1)	(4.1)	(8.7)	(4.2)	(4.4)
	PSw	22.6	19.8	20.4	21.3	13.8	16.9	17.2	16.3	21.4	23.5	25.3	22.1
		(10.4)	(9.1)	(10.3)	(11.0)	(11.3)	(16.2)	(12.0)	(14.4)	(9.9)	(9.3)	(12.0)	(9.9)
	Sw	15.6	12.9	13.5	14.3	16.5	15.4	13.5	12.8	16.9	19.3	19.4	19.8
		(10.1)	(6.8)	(8.3)	(8.1)	(10.3)	(8.3)	(7.3)	(4.4)	(3.5)	(7.1)	(7.2)	(7.1)
BF	LR	34.0	33.1	33.4	34.5	32.9	27.1	31.1	29.6	23.5	28.5	26.5	26.3
		(12.6)	(14.7)	(11.9)	(14.3)	(15.1)	(13.0)	(12.1)	(13.9)	(8.8)	(10.2)	(5.7)	(7.6)
	SS	18.4	20.4	21.3	21.2	24.5 ^c^	20.6	20.2	22.0	12.5 ^c^	15.0	13.5	14.0
		(9.4)	(12.9)	(14.3)	(10.9)	(14.0)	(11.8)	(12.2)	(13.9)	(7.0)	(7.5)	(5.0)	(6.3)
	PSw	9.9	10.0	10.0	8.3	16.2	16.2	15.0	16.4	7.5	8.6	8.3	8.8
		(7.9)	(9.6)	(7.1)	(5.2)	(12.1)	(17.7)	(10.8)	(13.4)	(5.9)	(6.7)	(7.6)	(6.3)
	Sw	20.7	17.1	18.6	18.4	25.6	23.2	24.7	23.9	23.7	26.5	27.2	26.2
		(11.9)	(6.7)	(8.1)	(7.1)	(11.0)	(10.5)	(11.1)	(10.8)	(8.7)	(7.6)	(8.3)	(6.6)

Values are presented as mean (standard deviation). Comparison of values at 1 min between the groups: paretic versus nonparetic, ^a^ *p* < 0.05; paretic versus control, ^b^ *p* < 0.05; nonparetic versus control, ^c^ *p* < 0.05 (Bonferroni test). Comparison over time within groups; ^†^ for 1 min, *p* < 0.05; ^‡^ for 6 min, *p* < 0.05 (Bonferroni test). LR, loading response; SS, single support; PSw, pre-swing; Sw, swing; TA, tibialis anterior; Sol, soleus; RF, rectus femoris; BF, biceps femoris.

## Data Availability

The data presented in this study are available on request from the corresponding author. The data are not publicly available due to privacy.
